# Association of health literacy, COVID-19 threat, and vaccination intention among Brazilian adolescents*

**DOI:** 10.1590/1518-8345.6154.3759

**Published:** 2022-11-07

**Authors:** Sidiany Mendes Pimentel, Marla Andréia Garcia de Avila, Rafaela Aparecida Prata, Hélio Rubens de Carvalho Nunes, Juliana Bastoni da Silva

**Affiliations:** 1 Universidade Federal do Tocantins, Palmas, TO, Brazil.; 2 Universidade Estadual Paulista “Júlio de Mesquita Filho”, Faculdade de Medicina de Botucatu, Botucatu, SP, Brazil.

**Keywords:** Health Literacy, Vaccination Refusal, COVID-19, Adolescent, COVID-19 Vaccines, Adolescent Health

## Abstract

**Objective:**

investigate the influence of health literacy on the assessment of COVID-19 threat to health and the intention not to be vaccinated among Brazilian adolescents.

**Method:**

cross-sectional study with 526 Brazilian adolescents aged 14 to 19 years. Socioeconomic aspects, health-disease profile, health literacy, health threat by COVID-19 and intention not to be vaccinated were analyzed by bivariate association and multiple linear regression with Poisson response.

**Results:**

higher health literacy score (p=0.010), cardiovascular disease (p=0.006), lower income (p=0.000), and living in the North region (p=0.007) were factors that contributed to feeling more threatened by COVID-19. Health literacy did not influence the intention not to be vaccinated (p=0.091), whose prevalence was lower among adolescents in the Southeast region when compared to those in the North region (p=0.010), among those who attended higher education (p=0,049) and those with higher income (p=0.000).

**Conclusion:**

health literacy influenced the perception of COVID-19 threat, but not the intention not to be vaccinated. Assessment of COVID-19 threat to health and prevalence of the intention not to be vaccinated were influenced by the region of residence, income, and education, which reinforces the importance of social determinants of health in this context.

## Introduction

The COVID-19 pandemic was declared by the World Health Organization in early 2020^1^ and, since then, in addition to the adoption of preventive measures such as hand sanitization, use of face masks in public places, and social distancing, the population had to acquire information about health to adapt their behaviors and avoid transmission^1^ ^-^ ^2^. Adolescents were considered a target group in COVID-19 transmission due to their form of socialization and group activities, and an important group, considering their susceptibility to the impacts of control measures, such as closing of schools, universities, and recreational areas, which were necessary restrictions in the onset of the pandemic^2^ ^-^ ^3^. Despite recognized relevance of social isolation measures, they also contributed to an increase in cases of mental health and domestic violence problems in this group^4^.

In addition, the literature shows some adolescents will also present health risk behaviors, a fact that deserves attention because, according to a national study, this group of the population seeks health care services at lower frequencies when compared to other age groups^5^ ^-^ ^6^.

Adolescence is a period marked by complex physical and social changes^7^. It has three stages: early adolescence (10 to 14 years old), middle adolescence (14 to 17 years old), and late adolescence (17 to 20 years) when major changes in autonomy are observed^8^.

In adolescence, health literacy (HL) is relevant to the adoption of disease prevention practices. HL is a field under construction, of a complex, multidimensional and interdisciplinary nature. It is defined as the ability to obtain, process, and understand health information and use such knowledge to make proper health decisions and adhere to treatment^9^. Health literacy has been identified as the new vital sign and a modifiable social determinant of health^10^ ^-^ ^12^, which can promote adherence to healthy behaviors in adolescence^13^.

Despite the few studies about HL in Brazil, studies conducted in other countries, such as China^14^, South Korea^15^, and Norway^16^, show the impact on the lives of adolescents, with evidence of individuals with low HL and less knowledge about their clinical problems, a higher number of hospital admissions, higher costs, and worse health status when compared to people with higher HL^17^ ^-^ ^19^. A recent investigation, which analyzed 17 studies from different countries, found scientific evidence that low levels of HL are associated with overweight among children and adolescents and initiatives to improve HL levels, in this case also among parents, can contribute to the management of obesity^20^.

A systematic review confirms the positive relationship between higher levels of HL and better health outcomes among adolescents^21^. On the other hand, studies conducted in Türkiye^22^ and Italy^23^ during the pandemic showed that low levels of HL are associated with higher rates of vaccine hesitancy.

A pioneering cross-sectional study with adolescents about COVID-19 vaccine hesitation was conducted in four cities in China. The mean age of the adolescents was 14.2 years, and 31.6% of them were hesitant to be vaccinated and 8.4% were resistant to vaccination. In this study, vaccine hesitancy among Chinese students was associated with limited HL and lower awareness of the risk of being infected by COVID-19^24^.

Adolescence is a strategic period of life for the identification of HL and behaviors that can influence health, because such knowledge will support the planning of actions focused on adolescents, the development of healthy lifestyles that, in turn, time, are important for future healthy adults^25^.

Then, this study aimed to investigate the influence of health literacy on the assessment of COVID-19 threat to health and the intention not to be vaccinated among Brazilian adolescents.

## Method

### Study design, scenario, and period

This is an exploratory cross-sectional study conducted with Brazilian adolescents from the five macro-regions of the country. It was conducted in Brazil with around 18,452,517 adolescents^26^. This study used a tool named Strengthening the Reporting of Observational Studies in Epidemiology (STROBE)^27^ to ensure the study method quality.

Data were collected online, from July 13 to September 30, 2021, using Google Forms. Participants were predominantly recruited using the snowball technique^28^. The study form was distributed via social media platforms (Instagram, Facebook, Twitter, TikTok, and Kwai), digital communication platforms (WhatsApp, Gmail, and phone calls), and to personal contacts of the authors (emails and WhatsApp). Schools, universities, churches, municipal and state health and education departments from all five macro-regions of Brazil helped with form distribution by sharing the invitation on social media and sending it to communication groups with parents and students.

### Sample and selection criteria

The sample consisted of individuals in the stages of middle or late adolescence, aged 14 to 20 years (incomplete), i.e., 19 years, 11 months, and 29 days. Inclusion criteria were adolescents aged 14 to 19 years with internet access. Exclusion criteria were adolescents whose forms were incomplete.

For the sample calculation, the sampling theory used non-probability sampling with intentional method, that is, participants were not referred to our study, but accepted to participate after becoming aware of the study through any of the platforms used to share it^29^. Sample calculation also considered: 1) that the eight questions of the health literacy questionnaire validated in 2017^30^ can be used to build a scale to quantify health literacy; 2) that the prevalence of intention not to be vaccinated was 14.8% among adolescents with low HL scores and 7.4% among adolescents with high HL scores^16^; 3) type I and II errors were defined as equal to 0.05 and 0.10, respectively; 4) the presence of nine confounding variables; and 5) simple random sampling. Given these assumptions, the sample size calculation resulted in 526 adolescents to analyze the association between HL and the intention not to be vaccinated. The sample size was calculated considering a comparison between two binomial proportions from independent samples and the addition of 15 subjects to each confounding variable inserted in the multiple regression model. Data collection was interrupted after 526 Brazilian adolescents completed the study instruments, which included participants from 25 Brazilian states and the Federal District. Only the state of Amazonas was not represented in this study.

### Study instruments

Sociodemographic and health-disease data were collected using a form with eight items developed by the authors based on social indicators used by the Brazilian Institute of Geography and Statistics (IBGE)^31^, which gathered information about the state of residence, age, sex, income, education, history of diseases and hospitalizations, and medications in the last six months.

The questionnaire used to assess the COVID-19 threat to health and the intention to be vaccinated was adapted from a study conducted in 2021^16^. It had four questions. The first three addressed the adolescent’s judgment of COVID-19 threat to health, with answers in a modified Likert scale, as follows: totally disagree, disagree, agree and totally agree (total score of 3 to 12). The fourth question was divided into two items and addressed: 1) the COVID-19 vaccination status (I have not been vaccinated so far, I received one dose, I received two doses, I received a single dose vaccine); and 2) intention to be vaccinated, for those not yet vaccinated or vaccinated with one dose, with five response options ranging from extremely unlikely, somewhat unlikely, not sure, somewhat likely, and extremely likely. For analysis purposes, the answers to the question about the intention to be vaccinated were divided as follows: respondents who answered “extremely likely” were considered as likely to be vaccinated and those who marked the other answers were considered as likely not to be vaccinated.

Data collection regarding HL used Health Literacy Assessment Tool, Portuguese version (p-HLAT-8), which has been validated^30^. The original instrument was developed by researchers from Switzerland to understand different dimensions of HL in the context of family and friends (close people) using a short instrument^32^. The Brazilian version was tested with 472 Brazilian university students and showed reliable results, allowing the calculation of an overall health literacy score, considering the proper weight for each item. The tool consists of eight questions with answers organized on a Likert scale ranging from 0 to 5 points. The questions assess: (1) the understanding of health information (questions 1 and 2 - 10 points); (2) search for health information (questions 3 and 4 - 8 points); (3) interactivity in health care (questions 5 and 6 - 10 points); and (4) critical health knowledge (questions 7 and 8 - 9 points). The overall p-HLAT-8 score ranges from 0 (worst score) to 37 points (best/ideal score) and there is no definition of a cut-off point, nor a HL classification as satisfactory or unsatisfactory. According to this instrument, higher scores indicate higher HL of the respondent.

### Study variables

#### Independent variable

Health literacy measured by p-HLAT-8^30^ ^)^ (0 to 37 points).

#### Potential confounders

The confounding variables were sociodemographic characteristics, such as sex (male and female), region of residence (North, Northeast, Midwest, Southeast, South), age (in years), education (elementary, high school, higher education), and family income (number of minimum wages), health-disease profile regarding the presence of chronic disease (yes or no), type(s) of chronic diseases (specified by the participant and categorized by the authors), hospital admission in the last six months (yes or no), and medication use (yes or no). Adjustments were made to multiple regressions to control confounders (Tables 3 and 5) and test the impact of HL considering the effect of confounding variables previously identified through bivariate associations (Tables 2 and 4).

#### Outcomes

The outcome variables were COVID-19 threat to health (3 to 12 points) and intention not to be vaccinated against the disease (yes/no).

### Data analysis

For the analysis, Poisson models and classical regression models were used, considering that Poisson models can be adopted to test associations through the prevalence ratio in cross-sectional epidemiological studies with a binary variable (in this case, the outcome variable of vaccination intention), while classical regression models with normal response can be adopted to test associations in cross-sectional studies with numeric variable, like the other outcome variable of this study (COVID-19 threat score)^33^.

Data analysis was performed in two phases: in the first phase, simple linear regression models were adjusted (with Poisson response for the vaccination intention outcome and normal response for the COVID-19 threat outcome) to estimate the association of every variable individually with the outcomes. Variables with p<0.20 association were added to a multiple linear regression model (Poisson response for the vaccination intention outcome and normal response for the COVID-19 threat outcome). In the multiple linear regression models, for every outcome, the relationships that presented p<0.05 were considered statistically significant. Analyses were performed with SPSS version 21.

### Ethical aspects

This study was approved by the Research Ethics Committee of the institution in charge, with Certificate of Submission for Ethical Analysis nº 48257321.0.0000.5519 and report nº 4.833.554/2021. Acceptance to participate in the study was confirmed with an informed consent term signed by adolescents aged 18 years or older or parents/guardians in case of adolescents under 18 years old. Adolescents under 18 old used the digital version of the informed assent form to agree with the study participation. Participants also had a chance to choose whether to receive a copy of their answers and the study results.

## Results

A total of 528 adolescents participated in the study, but two were excluded due to incomplete questionnaire responses, resulting in a final sample of 526 adolescents. Of these, 49% (n=258) were from the North region, 36.3% (n=191) from the Southeast, 6.8% (n=36) from the Midwest, 5.9% (n=31) from the Northeast, and 1.9% (n=10) from the South region. The states with more participants were Tocantins (46%, n=242), São Paulo (28.5%, n=150), Minas Gerais (4.9%, n=26), and Goiás (3.8%, n=20).

According to Table 1, the mean age of the adolescents was 16.9 years (sd±1.6), most were female, living in the North region, attending or having completed high school, and belonging to families with 2 to 5 minimum wages. About 9.0% (n=49) of the adolescents reported living with a chronic disease, with the prevalence of cardiovascular disease; 4.0% of the participants (n=21) had been hospitalized recently (in the last six months), and about 19.0% (n=102) reported using some medication.

**Table 1:** Sociodemographic characterization and health-disease profile of Brazilian adolescents (n=526). Brazil, 2021

Characteristic	Total	
	n	%
Sex		
Female	356	67.7
Male	170	32.3
Age	16.93^*^	1.6^†^
Region		
North	258	49.0
Northeast	31	5.9
Midwest	36	6.8
Southeast	191	36.3
South	10	1.9
Schooling level		
Elementary	67	12.7
High school	353	67.1
Higher education	106	20.2
Income (number of minimum wages)		
<0.5	116	22.1
0.5-1	52	9.9
1-2	136	25.9
2-5	141	26.8
5-10	45	8.6
10-20	26	4.9
>20	10	1.9
Presence of chronic disease		
No	477	90.7
Yes	49	9.3
Description of chronic disease		
Cardiovascular	22	4.2
Respiratory	5	1.0
Integumentary	3	0.6
Endocrine	3	0.6
Other	16	3.04
Recent hospitalization		
No	505	96.0
Yes	21	4.0
Medication use		
No	424	80.6
Yes	102	19.4

^*^Mean; ^†^standard deviation

Regarding the assessment of the COVID-19 threat to health, the mean was 7.7 points (SD±2.2), with a median of 8.0 (min. 3 and max. 12). For health literacy, the mean was 25.3 points (SD±5.4), with a median of 26.0 (min. 0 and max. 37).

The vaccination status for COVID-19 was: 65.2% (n=343) of participants had not received any dose, 24.9% (n=131) had already received the first dose, and 9.9% (n=52) had received the two doses of the vaccine or the single dose vaccine.

The intention to receive the vaccine against COVID-19 was assessed among participants not yet vaccinated, or who had only received the first dose; 86.9% (n=457) of them showed intention to be vaccinated. Table 2 shows data of the bivariate analysis, which assessed the relationship between sociodemographic variables, variables related to the health-disease profile, HL, and the dependent variable of “assessment of COVID-19 threat to health.”

**Table 2:** Bivariate analysis for the assessment of COVID-19 threat to health among Brazilian adolescents (n=526). Brazil, 2021

Characteristic	b*	95%CI^†^		p^‡^
Female participants	0.024	−0.385	0.434	0.907
South region	0.881	−0.519	2.282	0.217
Southeast region	−0.573	−0.988	−0.158	**0.007**
Midwest region	−0.391	−1.164	0.382	0.322
Northeast region	0.210	−0.615	1.036	0.617
North region^§^				
Age	0.035	−0.084	0.153	0.565
Higher education	0.359	−0.326	1.043	0.304
High school	0.154	−0.431	0.738	0.606
Elementary^||^				
Income	−0.263	−0.386	−0.139	**0.000**
Chronic disease	0.789	0.134	1.444	**0.018**
Cardiovascular disease	1.628	0.682	2.574	**0.001**
Recent hospitalization	1.043	0.069	2.017	**0.036**
Medication use	0.271	−0.213	0.755	0.272
Health literacy	0.041	0.006	0.076	**0.021**

^*^Intercept; ^†^Confidence interval; ^‡^Bivariate analysis by simple linear regression with Poisson response; ^§^Reference for the regions of Brazil; ^||^Reference for schooling levels

The multivariate analysis presented in Table 3 shows that adolescents in the Southeast region felt less threatened by COVID-19 when compared to those in the North region of the country (p=0.007). Also, the higher the income, the lower the COVID-19 threat, as perceived by the adolescents (p=0.000). Higher health literacy (p=0.010) and the presence of a cardiovascular disease (p=0.006) contributed to adolescents feeling more threatened by COVID-19. The score representing the perception of Brazilian adolescents of COVID-19 threat to health was, on average, 1.6 higher among participants with a cardiovascular disease versus those without this condition. Also, one point higher in HL generated an average increase of 0.044 points in the assessment of COVID-19 threat to health.

**Table 3:** Multivariate analysis to determine the factors associated with the perception of Brazilian adolescents of COVID-19 threat to health (n=526). Brazil, 2021

Characteristic	b^*^	95%CI^†^		p^‡^
South region	1.038	−0.316	2.391	0.133
Southeast region	−0.567	−0.976	−0.157	**0.007**
Midwest region	−0.244	−0.993	0.505	0.524
Northeast region	0.056	−0.743	0.855	0.891
North region^§^				
Income	−0.239	−0.361	−0.117	**0.000**
Chronic disease	0.028	−0.810	0.866	0.948
Cardiovascular disease	1.685	0.479	2.891	**0.006**
Recent hospitalization	0.803	−0.142	1.747	0.096
Health literacy	0.044	0.010	0.078	**0.010**

^*^Intercept; ^†^Confidence interval; ^‡^Multiple Linear Regression with Poisson response; ^§^Reference for the regions of Brazil

Table 4 shows data of the bivariate analysis, which assessed the relationship between sociodemographic variables, the health-disease profile, and HL on the outcome of intention not to be vaccinated.

**Table 4:** Bivariate analysis for the assessment of the intention not to be vaccinated among Brazilian adolescents (n=526). Brazil, 2021

Characteristic	b^*^	95%CI^†^ b		PR^‡^	95%CI PR		p^§^
Female participants	−0.088	−0.600	0.425	0.92	0.55	1.53	0.738
South region	−0.641	−2.621	1.338	0.53	0.07	3.81	0.525
Southeast region	−1.394	−2.105	−0.683	0.25	0.12	0.51	**0.000**
Midwest region	−0.313	−1.233	0.607	0.73	0.29	1.84	0.505
Northeast region	−0.163	−1.084	0.757	0.85	0.34	2.13	0.728
North region^||^							
Age	−0.304	−0.449	−0.158	0.74	0.64	0.85	**0.000**
Higher education	−3.167	−5.191	−1.143	0.04	0.01	0.32	**0.002**
High school	−0.400	−0.973	0.174	0.67	0.38	1.19	**0.172**
Elementary education^¶^							
Income	−0.462	−0.643	−0.282	0.63	0.53	0.75	**0.000**
Chronic disease	−0.512	−1.522	0.497	0.60	0.22	1.64	0.320
Cardiovascular disease	−1.088	−3.062	0.886	0.34	0.05	2.43	0.280
Recent hospitalization	0.089	−1.068	1.246	1.09	0.34	3.48	0.880
Medication use	−0.756	−1.538	0.025	0.47	0.21	1.03	**0.058**
Health literacy	−0.054	−0.090	−0.018	0.95	0.91	0.98	**0.003**

^*^Intercept; ^†^Prevalence ratio; ^‡^Confidence interval; ^§^Bivariate analysis by simple linear regression with normal response; ^||^Reference for the regions of Brazil; ^¶^Reference for education levels

Table 5 shows the prevalence of the intention not to be vaccinated was lower among adolescents in the Southeast region when compared to those in the North region (p=0.010). Likewise, the prevalence of the intention not to be vaccinated was lower among adolescents who attended higher education when compared to those whose education level was elementary school (p=0.049). In addition, the higher the income, the lower the intention not to be vaccinated (p=0.000). Although the bivariate analysis showed an association between HL and the intention not to be vaccinated, this relationship was not confirmed in the multivariate analysis.

**Table 5:** Multivariate analysis to determine the factors associated with the intention not to be vaccinated among Brazilian adolescents (n=526). Brazil, 2021

Characteristic	b^*^	95%CI^†^ b		PR^‡^	95%CI PR		p^§^
Intercept	2.426	−0.817	5.669	11.32	0.44	289.88	0.143
South region	−0.477	−2.484	1.530	0.62	0.08	4.62	0.641
Southeast region	−0.974	−1.715	−0.234	0.38	0.18	0.79	**0.010**
Midwest region	−0.162	−1.096	0.773	0.85	0.33	2.17	0.734
Northeast region	−0.445	−1.380	0.489	0.64	0.25	1.63	0.350
North region^||^							
Age	−0.154	−0.361	0.052	0.86	0.70	1.05	0.143
Higher education	−2.219	−4.426	−0.011	0.11	0.01	0.99	**0.049**
High school	−0.029	−0.757	0.699	0.97	0.47	2.01	0.938
Elementary education^¶^							
Income	−0.376	−0.562	−0.190	0.69	0.57	0.83	**0.000**
Recent hospitalization	−0.379	−1.171	0.414	0.68	0.31	1.51	0.349
Absence of recent hospitalization	0^a^						
Health literacy	−0.033	−0.071	0.005	0.97	0.93	1.01	0.091

^*^Intercept; ^†^Confidence interval; ^‡^Prevalence ratio; ^§^Multiple linear regression with normal response; ^||^Reference for the regions of Brazil; ^¶^Reference for education levels

## Discussion

In our study, higher HL was associated with adolescents feeling more threatened by COVID-19. Also, one point higher in HL generated an average increase of 0.044 points in the assessment of COVID-19 threat to health.

COVID-19 threat to health showed an average score of 7.7, which is close to the midpoint of the scale used (score range from 3 to 12). A similar behavior was observed in a North American study that assessed individuals in late adolescence, adults, and older adults, in which the assessment of COVID-19 threat to health resulted in an average score of 3.13 points on a scale of 1 to 5 points, also close to the midpoint of the scale used by the authors^16^. Despite the age differences between the participants of our study and the North American study^16^, the assessment of COVID-19 threat to health was similar between the samples of both studies.

Health literacy ensured a better understanding of COVID-19 and its risks to health, which can favor positive choices, as recommended by health professionals and organizations, a result agrees with those from other studies^1^ ^,^ ^15^ ^-^ ^16^. In this sense, professionals from health care systems should invest in health education actions to enhance population HL, including debates about diseases and how to avoid transmission. Also, communication and strategies appropriate to the target audience should also be considered.

In general, health education develops in individuals a responsibility for their own health and the collective health. And today, its social determinants must also be considered, as it happened before the Brazilian health reform movement^34^ ^-^ ^35^.

The influence of social determinants of health on the assessment of COVID-19 threat to health and the prevalence of the intention not to be vaccinated were observed in the results of our study, as the outcomes were influenced by income, education, and the region of Brazil where the adolescent lives.

Adolescents in the Southeast region felt less threatened by COVID-19 when compared to those in the North region. Also, higher income indicated adolescents felt less threatened by COVID-19. These findings are justified in the literature, considering that social determinants, such as working and living conditions, directly affect the population health. Although Brazil has improved its health indicators in recent decades, they are worse in the North region, just like social indicators^36^.

This study did not analyze the working and living conditions of the adolescents; however, when considering the social reality of the North region, we can infer that lower income leads to worse housing conditions, with more people living together, and, in general, people have informal jobs or work in essential services during the pandemic. These social issues contributed to the fact that adolescents in the North region were more vulnerable to contamination by SARS-CoV-2, as seen in COVID-19 mortality rate of this region, which was 84% higher than the national average (27.7/100,000 inhabitants)^37^ in 2020. Therefore, it may justify the stronger perception of COVID-19 threat to health among adolescents in this region.

In addition, adolescents with a cardiovascular disease felt more threatened by COVID-19. The scientific literature explains this perception, since people with chronic diseases tend to have more severe forms of COVID-19 and a higher prevalence and severity of symptoms, including dyspnea^38^ ^-^ ^40^.

Considering that COVID-19 is a real threat to the health of the population, which has killed 6,300,398^41^ people so far, vaccination against COVID-19 is recommended by the academic and scientific community for health protection for the general population, including children and adolescents^42^ ^-^ ^43^.

In our study, 86.9% (n=457) of the adolescents reported intention to be vaccinated against COVID-19, in agreement with a study conducted in Latin America and the Caribbean, in which around nine out of ten parents intended to vaccinate their children and adolescents against COVID-19^44^. In Brazil, according to an epidemiological bulletin published on March 8, 2022, vaccination coverage for the age group of 12 to 17 years, with at least one dose of the vaccine, was around 80%^45^.

Regarding the factors that influenced the intention not to be vaccinated, this study found that the prevalence of the intention not to be vaccinated was lower among adolescents in the Southeast region when compared to those in the North of the country, as well as among those who attended higher education versus those who had attended elementary school and among adolescents with higher income.

The association of a higher level of education of parents with a lower prevalence of intention not to vaccinate their children was also reported in a study with parents from Latin America and the Caribbean^44^. Likewise, a North American study of national coverage, which sought to identify predictors of the intention to vaccinate against COVID-19, also showed that high family income and higher education were associated with stronger vaccination intention^16^. Education and income are closely related, and improvements in these social determinants drive changes in lifestyle, which favor the promotion of health^46^, including vaccination against COVID-19.

In order to better understand vaccination adherence among adolescents, a study assessing adolescents from Acre, which is part of the Amazon region, investigated the acceptability of the vaccine for human papillomavirus (HPV), recently introduced in the vaccination schedule in Brazil. Only 46.1% of the adolescents who are part of the target population of the campaign were vaccinated. The study concluded that unvaccinated adolescents had knowledge gaps about the virus and its respective vaccine when compared to the group that received the HPV vaccine. Then, such information must be disseminated among adolescents, parents, and even among the health professionals^47^. These findings support reflections on the current challenge for the health system in Brazil to increase vaccination coverage in the country.

Despite the undeniable importance of providing information about health to the population, psychic, social and subjective factors, such as popular beliefs, can influence the way people handle issues of their daily life, not to mention the fake news, which makes the work of health professionals difficult and increases the population’s vulnerability to diseases. A recent study analyzed fake news about vaccines and COVID-19, highlighting that Brazil, with its educational problems, favors the dissemination of misinformation. Currently, one out five fake stories in Brazil is about vaccines, a situation that contributed to the non-adherence of part of the population to social distancing and vaccination campaigns^48^.

Regarding HL, the adolescents in our study presented mean HL score of 25.3 in the HLAT-8 instrument^30^. This score represents about 68% of the maximum HL score (37 points) using this scale. Other studies^49^ ^-^ ^51^ that used the same instrument showed similar HL scores to those of our study. Chinese adolescents^49^, mean age of 13.4 years, had a mean HL score of 26.3, a little higher than the score obtained in our study. In the period of the COVID-19 pandemic, HL score was 25.6^50^ among university students in China and 27.4^51^ among university students in Italy.

In our study, no statistically significant relationship was observed between HL variables and the intention not to be vaccinated; however, based on our analysis, the higher the HL score, the lower the prevalence of the intention not to be vaccinated among adolescents. In this sense, further studies on HL should be conducted with people in this phase of life, in order to contribute to the planning of the practice of health professionals who work directly with adolescents - or indirectly, such as those responsible for the formulation of public policies focused on adolescents.

Further studies about HL and health-related behaviors with adolescents are also justified considering the complexity of the topic. Possible interventions by nursing professionals should be studied and planned in order to improve HL and contribute to the adoption of healthier behaviors by adolescents. Faced with this challenge, nurses must develop interprofessional and intersectoral work in health, in partnership with the education and social service sectors, to increase the chances of achieving good results.

Participant selection bias was a study limitation, considering that part of the sample was initially constituted of contacts of researchers. Also, data collection was conducted online, which excluded the population without internet access. Then, more vulnerable adolescents without internet access were not represented in our study, which highlights the need for further studies that include this population using fieldwork methods, with face-to-face data collection. However, our study contributes to knowledge about HL among adolescents, a subject that is still not fully explored in the country. It also provides new data about how this indicator (HL) interferes in the adolescent’s perception of COVID-19 and their health-related choices, such as COVID-19 vaccination.

## Conclusion

The assessment of COVID-19 threat to health, from the perspective of Brazilian adolescents, was influenced by health literacy, region of residence, income, and presence of a cardiovascular disease. Factors such as region of Brazil, income, and education can impact the intention not to be vaccinated. The association between HL and intention not to be vaccinated was not statistically significant, but a trend was observed: higher HL scores generate a lower prevalence of intention not to be vaccinated. Our study provide unprecedented data for adolescent health in Brazil and highlight the importance of social determinants of health in this context, which must be considered by health professionals when planning, performing, and assessing their practices.
